# Recent advances in understanding the genetics of sleep

**DOI:** 10.12688/f1000research.22028.1

**Published:** 2020-03-27

**Authors:** Maxime Jan, Bruce F. O'Hara, Paul Franken

**Affiliations:** 1 Centre for Integrative Genomics, University of Lausanne, Lausanne, 1015, Switzerland; 2 Department of Biology, University of Kentucky, Lexington, 40515, USA

**Keywords:** Sleep, Sleep Regulation, Genetics, Genetic Screen, Forward genetics, GWAS, GRP

## Abstract

Sleep is a ubiquitous and complex behavior in both its manifestation and regulation. Despite its essential role in maintaining optimal performance, health, and well-being, the genetic mechanisms underlying sleep remain poorly understood. Here, we review the forward genetic approaches undertaken in the last four years to elucidate the genes and gene pathways affecting sleep and its regulation. Despite an increasing number of studies and mining large databases, a coherent picture on “sleep” genes has yet to emerge. We highlight the results achieved by using unbiased genetic screens mainly in humans, mice, and fruit flies with an emphasis on normal sleep and make reference to lessons learned from the circadian field.

## Introduction

Through genetic analyses of sleep, we hope to learn about the molecular substrates of this remarkable behavior and its pathologies. Sleep is remarkable because we spend a considerable portion of our lives in this state (>24 years for most of us) yet we know little about its likely numerous functions. Clearly, sleep is important as lack of sleep, poor-quality sleep, or sleep disorders greatly impact performance and well-being and may lead to poor health. Genetics can be used to approach a variety of questions about sleep regulation, sleep function, abnormal sleep, and the consequences of poor or insufficient sleep. For example, research aimed at discovering the gene variants altering susceptibility to the deleterious effects of sleep loss and the molecular pathways activated by sleep loss are two areas of great interest, from both a basic scientific understanding and a clinical point of view.

With perhaps some envy, sleep geneticists look at the astounding progress made in the closely related circadian rhythms research field. Results from mutagenesis screens in flies and mice for circadian phenotypes ^1, 2^, mostly variations in circadian period length, led to the discovery of a core set of interacting genes (that is, “clock genes”) that pace the clock ^3^ and to a Nobel prize in 2017 ( www.nobelprize.org/prizes/medicine/2017/prize-announcement/). With this promise, mutagenesis screens have been applied to the problem of sleep, and although important new insights have been gained, a core set of genes regulating sleep (apart from those involved in sleep’s circadian modulation) remains to be identified. Perhaps one reason hampering breakthrough progress matching that in the circadian field is that sleep is a circuit-driven behavior whereas circadian rhythms rely on cell-autonomous processes and thus are more tractable in terms of identifying key players through genetic dissection. Moreover, sleep is not one phenotype but a multi-faceted behavior starting off with two very different sleep states—rapid eye movement (REM) sleep and non-REM (NREM) sleep—that seem preserved across vertebrates ^4^ and that each have their distinct (neuro)physiology, regulation, and functions. In a recent study, we quantified well over 300 sleep-related phenotypes in a genetic reference population (GRP) of mice ^5^. These phenotypes included time spent in the various sleep-wake states, their distribution over the day, and the frequency- and amplitude-specific characteristics of the electroencephalogram (EEG), all under undisturbed baseline conditions and after a period of enforced waking. The median heritability in this comprehensive sleep-wake phenome was high (h ^2^ = 0.68), and although a number of phenotypes were highly correlated, many among them proved to be independent sleep traits each with their distinct polygenic mode of inheritance. It is therefore unrealistic to aim at finding core “sleep genes”; instead, we expect to uncover sets of genes or gene pathways each modulating specific aspects of sleep. Such genes could affect sleep traits directly or could do so indirectly through early (or late) processes of neurodevelopment and neuroplasticity. In addition to its phenotypic complexity, sleep varies with life history and environment and can differ greatly among animal species according to their ecological niche. Therefore, genetic analysis of sleep in species other than the established model species that have been used thus far could help delineate conserved genetic pathways regulation sleep ^6^.

With this in mind, this brief overview of the progress in the field will focus on the recent efforts to discover the genetic underpinnings of sleep in various species and mainly concern flies, mice, and humans with some reference to zebrafish and *Caenorhabditis elegans* work. Genetics is a broad term and is used differently among fields; for example, we will have to ignore optogenetics although this technique has revolutionized sleep research in the last 15 years in dissecting its underlying neuro-circuitry ^7, 8^. Similarly, we will not be comprehensive in reporting on the ever-growing list of candidate genes that have been genetically targeted to study their specific role in aspects of sleep, although much has been learned using this approach. This “gene-to-phenotype” approach is referred to as reverse genetics. In reply, classic genetic approaches (from phenotype to gene), which include the mutagenesis screens mentioned above, have become known as forward genetic approaches. However, these methods can clearly overlap and the distinction depends on scale; a large screen of knockout mice for known single genes could be considered a large-scale reverse genetic approach but is really more similar to a mutagenesis screen, as we will discuss below. Here, progress in the last four years (2016–2019) using unbiased gene discovery approaches will be reviewed with a strong emphasis on studies focusing on “normal” sleep and its regulation as opposed to sleep-related disorders.

## Forward genetic screens for induced genetic variants

In forward genetic screens, changes in gene function are induced more or less randomly ^9^ throughout the genome. Mutations affecting gene function have been made through the use of chemicals—for example, N-ethyl-N-nitrosourea (ENU) or ethyl methanesulfonate (EMS)—or, in the fruit fly, *Drosophila melanogaster* through insertion of transposable P elements. Screens for the effects of overexpression of genes can be achieved by making use of the GAL4-upstream activation sequence (GAL4-UAS) system ^10^ or through injection of plasmids containing inducible overexpression vectors ^11^. Phenotyping of a large number of animals will identify lines with aberrant phenotypes caused by single genes.

The earliest sleep screen was performed in *Drosophila* ^12^. In that study, about 9,000 mutagenized lines were phenotyped for extreme short sleep time. Flies carrying loss-of-function mutations for *Shaker*, a gene encoding a voltage-dependent potassium channel, were found to sleep exceptionally little. Surprisingly, after screening several thousand additional mutagenized lines, a later *Drosophila* sleep screen identified the gene *Sleepless*, which proved to be a regulator of *Shaker* ^13^. Together, the two studies suggest neuronal membrane excitability as a core feature of homeostatic sleep drive ^13, 14^.

In mammals, the first mutagenesis sleep screen was published in 2016 ^15^. ENU was used to induce random point mutations as 8,000 mice were screened for sleep abnormalities. A mutation in a single phosphorylation site in the protein kinase *Sik3* was found to increasehomeostatic sleep drive concomitantly with increased time spent in NREM sleep ^16^. SIK3 was found to associate specifically with phosphoproteins for which the phosphorylation state was found to be sleep-wake–driven, suggesting that the sleep homeostatic process represents a phosphorylation (during wakefulness)/dephosphorylation (during NREM sleep) cycle ^17, 18^. Moreover, in the same screen, a missense mutation in *Nalcn*, a voltage-independent and non-selective cation channel, reduced time spent in REM sleep, and a point mutation in *Cacna1a*, encoding a voltage-dependent calcium channel, reduced wakefulness by about 70 minutes per day ^19^. While other genes discovered with this screen are likely to be in the pipeline, the yield thus far seems disappointing given the large number of mice phenotyped.

In *C. elegans*, forward genetic screens for sleep have been performed as well. In these nematodes, periods of sleep can be observed at the transition between developmental stages (developmentally timed sleep) or sleep can be induced by stress (stress-induced sleep) ^20^. Upon stress, the release of the neuropeptide Flp-13 (FMRFamide-like peptide-13) was shown to mediate the sleep-promoting effect ^21^. In an EMS mutagenesis screen in Flp-13–overexpressing worms for genes modifying the sleep-promoting effect of Flp-13, its receptor *Dmsr-1*, encoding a G protein–coupled receptor, was identified ^22, 23^. The G-protein that is coupled to Dmsr-1 may be the G _i/o_ alpha subunit *Goa-1*, which was identified in another mutagenesis screen for genes regulating developmentally timed sleep, along with *Gpb-2*, encoding another G-protein subunit ^24^. Together, these results confirm the importance of G-protein signaling pathways in worm sleep.

Screens in which the effects of gene overexpression on sleep were studied were performed in *Drosophila* and zebrafish. By phenotyping about 12,000 fly lines for sleep duration *Nemuri*, encoding an uncharacterized antimicrobial peptide, was identified, which when overexpressed in adults induced sleep, thereby linking sleep and the immune system ^10^. In a zebrafish-overexpression screen for secreted proteins, the neuropeptide Neuromedin U ( *Nmu*) was identified to regulate sleep-wake behavior ^11^. Interestingly, anatomical and functional analyses found that *Nmu*-induced arousal is mediated by corticotropin-releasing hormone (CRH) signaling in the brainstem specifically, independent of the role of CRH in the hypothalamic–pituitary–adrenal axis. Also, overexpression of the neuropeptide Neuropeptide Y (NPY) in this screen was found to affect sleep and the authors could establish that this effect was mediated through inhibition of noradrenergic signaling known to promote wakefulness ^25^.

The ongoing efforts of the International Knockout Mouse Consortium (IKMC) to “knock out” each protein-coding gene and of the International Mouse Phenotyping Consortium (IMPC) to determine the phenotypic consequences ^26^ should be mentioned here because this (in principle) “reverse genetic” approach must be considered “forward” given its generally unbiased and genome-wide character, as already pointed out above. Sleep is part of the IMPC phenotyping pipeline and thus far 512 null-mutant strains have been evaluated; 72 of these displayed aberrant values for some of the 13 sleep-related variables quantified ( www.mousephenotype.org). These results suggest that a high percentage of gene products influence sleep in some way, including breathing rates during sleep ( https://doi.org/10.1101/517680). Several of the genes identified, such as *Ppp1r9b*, *Pitx3*, *Ap4e1*, and *Myh1*, affected time spent asleep to a similar degree as found in mutagenesis screens, suggesting that both mutagenesis and screens of knockout mice have found only a very small fraction of the genes that can influence sleep when mutated or eliminated. This again supports the notion that no core set of genes may underlie sleep regulation, at least to the same degree found for clock genes that underlie circadian rhythm generation. However, as discussed below, the transcriptional/translational oscillation of clock genes, especially outside the suprachiasmatic nuclei (SCN), appears to play a key role in sleep homeostasis as well (and a number of other functions).

The IKMC uses a combination of gene trapping and gene targeting in C57BL/6N mouse embryonic stem cells to mutate protein-coding genes. With CRISPR-Cas9, a more efficient technology has become available to target genes and might be used in genome-wide screens ^27^. Already, through the use of a triple-target CRISPR approach to generate bi-allelic knockouts in a single generation, targeting entire gene families has become feasible and applied to sleep (for example, acetylcholine receptors, N-methyl-D-aspartate receptors, and Ca ^2+^-dependent K ^+^ channels ^28– 30^) with some striking results; mice lacking the muscarinic acetylcholine receptors, *Chrm1* and *Chrm3*, were also found to lack REM sleep ^28^.

## Forward genetic mapping of natural genetic variants involved in sleep

The above-mentioned forward genetic screens report on the effects of single genes on a mostly uniform genetic background. However, many sleep traits are known to be genetically complex and each contributing gene usually explains but a small fraction of the overall phenotypic variance. Examples from the circadian field powerfully illustrate that genetic background matters. The first circadian clock gene identified in a mammalian mutagenesis was *Clock* ^2^. The ENU-induced *Clock* ^Δ19^ mutation discovered in that study caused circadian period lengthening and periodicity loss in mice of the C57BL/6J background but would have gone unnoticed if the melatonin-producing C3H/HeJ strain had been used in the screen instead ^31^. Moreover, linkage analysis of circadian traits quantified in a C57BL/6J × BALB/cJ - F2 population identified a number of quantitative trait loci (QTLs) that did not map to known clock genes ^32^, suggesting not only that many genes important in circadian rhythm generation await their discovery but also that some of the genes identified through mutagenesis might be invariant in natural populations and not contribute to phenotypic variability.

Although the phenotypic variance explained by QTLs underlying complex traits is generally small, which makes identifying the causative genes difficult, the above findings convinced sleep researchers of the necessity to study natural populations or population panels constructed through selective breeding, such as GRPs, to map the natural genetic variants and their interactions underlying differences in sleep traits. With this aim, genome-wide association (GWA) studies have been performed in flies, mice, and humans. Although the genetic contribution over environmental factors is generally high (h ^2^ >0.5 for many sleep-wake traits and h ^2^ >0.8 for a number of EEG-related traits ^5, 33, 34^), it arises from many possible genetic variants (so far, 650 million variations have been identified in human populations ^35^) and their interactions. This very large genotypic diversity and the small effect size of each necessitates sample sizes of several thousands to reach the *P* value threshold required to identify a variant significantly associated with a given trait (usually set to *P* <5 × 10 ^−8^), even when the interactions among them are ignored (epistasis ^36^). This is a problem for almost all normal and pathological traits that have been studied, including sleep. To address this problem, GRPs have been constructed in model species (see below).

The first “sleep” GWA study in humans was a case control study for the sleep-related disorder restless legs syndrome (RLS) and identified a number of genetic risk variants each conferring only minor increases in disease risk ^37, 38^. To date, six such RLS risk-conferring genomic loci have been found. Among those, functional contributions have been demonstrated in follow-up studies, including the use of mouse models, for the transcription factors *Meis1*, important for nervous system development and affecting dopamine signaling, and *Btbd9*, which sets the activity of medium spiny neurons in the striatum ^39– 42^.

GWA studies focusing on the quantitative aspects of normal sleep in the population rely on self-reported estimates of sleep and sleep problems through questionnaires or wrist actimetry devices or both. (Because of the large sample size required, classic EEG-based sleep phenotyping is time- and cost-prohibitive.) Variables such as sleep duration and timing, chronotype, sleep-onset latency, daytime sleepiness, or sleep problems (or a combination of these) have been assessed in an increasing number of studies by mining databases such as the UK Biobank ^43^ and 23andME combined or not with other large cohorts with aggregate sample sizes reaching more than 100,000 samples ^44– 54^. Many significant loci have been reported, some confirming results of other reports but all with quite small effect sizes (for example, explaining 3.5-minute differences in sleep duration) and without a coherent emerging picture explaining the large difference in sleep in the general population. The largest GWA analyses to date used more than 1.3 million individual samples to study insomnia complaints and found correlations able to explain 2.6% of the variance in insomnia ^55^. Many significant loci (202) were found pointing to about 1,000 genes that were identified through gene set enrichment analyses and available expression QTL (eQTL) and chromatin mapping data. Interestingly, overlap with RLS and RLS risk-conferring loci, including *Meis1* and *Btbd9*, and striatal medium spiny neurons were reported, pointing to shared etiology or to pleiotropic gene effects not uncommon in sleep and neurological disorders ^56^. Although computational approaches and the use of datasets on intermediate phenotypes (intermediate between genome and phenotype such as transcriptome and chromatin data) strengthen the likelihood that the identified candidate genes are causally involved, the challenge remains to experimentally demonstrate their biological relevance for the sleep phenotypes they associate with.

GWA studies have been performed in animals as well. For example, in a large cohort of CFW outbred mice, in which more than 5.7 million variants segregate, 19 aspects of sleep were quantified by using a piezoelectric device ^57^ as part of a larger phenotyping pipeline ^58^. QTLs for sleep quality containing single genes identified *Ppargc1a* and *Unc13c*, both of which are involved in synaptic plasticity and transmission ^59, 60^.

## Mapping of natural genetic variants underlying sleep traits in genetic reference populations

To better harness the genetic complexity contained in natural populations, mouse and fly GRPs have been created with a reduced but better-controlled and better-characterized genetic diversity. Fewer samples are needed to significantly associate sleep phenotypes to genotypes and to have the power to identify causal variants. In the fly, the *Drosophila*-GRP (DGRP), a set of about 200 recombinant inbred lines, was generated for the analyses of quantitative traits at a population level ^61^. A GWA study for sleep and activity phenotypes was performed with this GRP which yielded many single-nucleotide polymorphisms (SNPs) significantly associated with each trait, several of which mapped to genes over-represented in the epidermal growth factor receptor (Egfr) signaling pathway ^62^. Another GRP for *Drosophila* concerns a panel of 39 fully sequenced inbred lines selected for long and short sleep phenotypes: the Sleep Inbred Panel ^63^. In this panel, differences in sleep duration were associated with 126 polymorphisms pointing to 80 candidate genes related to the Egfr, Wnt, Hippo, and Mapk signaling pathways ^64^.

In the mouse, several GRPs have been derived from two or more isogenic strains such as the BXD ^65^, Collaborative Cross (CC) ^66^, Diversity Outbred (DO) ^67^, and Hybrid Mouse Diversity ^68^ panels. Some of these GRPs have been phenotyped for sleep. In the DO panel, moderate to high heritabilities for a number of sleep and circadian traits were observed by using a high-throughput phenotyping strategy ^69^. During construction of the CC panel, 50 sleep-related parameters and their response to sleep deprivation were assessed in about 600 “pre”-inbred mice (about F2:G5 or 75% inbred) carrying alleles from eight parental strains ^70^. For the resumption of activity after sleep deprivation, a significant and narrow QTL was identified encompassing two protein-coding genes ( *Ntm* and *Snx19*) involved in neural cell adhesion and insulin-containing vesicles maintenance, respectively. The BXD panel is perhaps the most widely used and best-characterized GRP. Earlier sleep analyses in the older BXD/ *TyJ* panel of recombinant inbred strains yielded *Homer1*, encoding a post-synaptic density scaffold protein involved in synaptic plasticity and calcium signaling, as a candidate gene for the increase in delta power, an EEG-derived proxy for sleep homeostatic drive, after sleep deprivation ^71, 72^. Additionally, retinoic acid receptor beta ( *Rarb*) was found to determine the contribution of slow waves to the NREM sleep EEG ^73^.

The QTLs identified in GRP panels can be quite large, encompassing many genes. Adding transcriptome data and other intermediate phenotypes such as proteome and metabolome data not only can help to select candidate genes located within QTLs but also can give insight into the flow of information from DNA to phenotype at the level of a population and how environmental challenges such as sleep deprivation alter this information flow. This multi-level, integrative approach has been termed systems genetics ^74^. Systems genetics approaches for sleep have been pioneered in the fly and mouse ^75, 76^. Sleep phenotyping and transcriptome analysis of four brain regions in a C57BL/6J × 129S1/SvImJ intercross identified gene expression networks underlying sleep regulation, such as an *Arc*-driven gene modules associated with EEG delta power ^77^. Systems genetics analyses have also been performed in the more recent BXD/ *RwwJ* GRP. In addition to the comprehensive sleep-wake phenome already mentioned above, cortical and liver transcriptome data and plasma metabolomic data were collected as intermediate phenotypes all assessed under undisturbed baseline conditions and after sleep deprivation ^5^. Sleep deprivation was found to extensively reshape the systems genetics landscape by altering 60 to 78% of the transcriptomes and the metabolome, and numerous genetic loci affected the magnitude and in some cases even the direction of change. Systems genetics analyses imply α-amino-3-hydroxy-5-methyl-4-isoxazolepropionic acid (AMPA) receptor trafficking and fatty acid turnover as substrates of the negative effects of insufficient sleep. Perhaps the most surprising finding concerned the implication of *Acot11*, an enzyme involved in fatty acid regulation in the liver, in determining the recovery of NREM sleep after sleep deprivation, demonstrating that peripheral systems can be crucial in explaining phenotypes that are generally considered to be controlled centrally. (“Sleep is of the brain, by the brain, and for the brain” ^78^.)

Furthermore, integrating gene expression data such as from the Genotype-Tissue Expression (GTEx) database ^79^ with GWA studies in humans facilitated the identification of the specific cell types implicated in insomnia (for example, medium spiny neurons ^55^), identification of eQTLs (for example, *Cluap1* associated with the number of nocturnal sleep episodes ^52^), and feature construction for machine learning methods (for example, 4,374 eQTLs identified for morningness behavior like *Nmur2*, a Neuromedin receptor ^80^).

## Novel insights into sleep using familial pedigrees

Studying familial pedigrees in which extreme phenotypes segregate can be highly informative in discovering rare mutations, likely to be missed in large-population GWA studies. This was powerfully illustrated with cases of the circadian sleep disorder familial advanced sleep phase syndrome (FASPS), which led to the discovery of causal mutations in the core clock genes *Period 2* ( *Per2*) and *Casein kinase I delta* ( *Csnk1d*) ^81, 82^. Subsequently, a number of studies identified additional gene mutations affecting chronotype in humans, such as *Timeless (*Tim), Cryptochrome 2 (Cry2), and Period 3 ( *Per3*) (reviewed in ^83^). Many of the rare mutations identified had in common that they were of high penetrance and of large effect size which is a prerequisite for functional studies in model species such as fly and mouse, as illustrated in the next examples concerning sleep.

Besides the timing of sleep, which is generally considered a circadian phenotype (but see 84), a number of pedigrees in which a short sleep phenotype segregates have been reported. The first such study concerned the discovery of a mutation in *Dec2* conferring short sleep in humans and mice ^85^. Besides its role in circadian timekeeping ^86^, *Dec2* was found to act as a transcriptional repressor of *Hypocretin* ( *Hcrt*) encoding the two neuropeptides HCRT1 and 2, which are important in sleep-wake regulation ^87^. In another family with natural short sleepers, the causative mutation was found in the β1-adrenergic receptor ( *Adr1*), leading to decreased protein stability ^88^. Results in mice carrying the same mutation also showed a short sleep phenotype, albeit less pronounced compared with humans, and established an important role of β1-adrenergic receptors in sleep-wake regulation ^88^. A final example concerns a missense mutation in the G protein–coupled *Neuropeptide S receptor 1* ( *Npsr1*), also found to be associated with a natural short sleep phenotype in humans ^89^. Mice expressing the homologous mutations slept less and interestingly were resistant to the memory deficits usually associated with sleep loss, thus suggesting that the NPS/NPSR1 pathway determines sleep duration and links sleep homeostasis and memory consolidation ^89^.

## Discussion

In the above sections, we briefly outlined recent progress in the field of sleep genetics according to the different approaches used because a coherent picture on the core genes or gene pathways regulating specific aspects of sleep has yet to emerge. Sleep’s phenotypic complexity and the presumed redundancy of molecular pathways regulating specific aspects of sleep might be at the base of this. To some extent, such redundancy is also apparent at the neuro-anatomical level; a singular sleep center does not seem to exist that, when lesioned, eliminates sleep analogous to the effect of lesioning the SCN, which results in the elimination of circadian organization for most overt behaviors. To carry this analogy further, eliminating core clock genes (or combinations thereof) will render animals arrhythmic ^90^, while no genes for which null mutants preclude sleep are known. This might indicate that lacking sleep is embryonic lethal as long-term sleep deprivations are for adult rats and flies ^91, 92^ (but also see 93). In this respect, the finding of an all-but-complete abolishment of the EEG manifestation of REM sleep in mice lacking *Chrm1* and *3* is both surprising and encouraging ^28^.

The field of sleep genetics might profit by focusing on core aspects of sleep, such as its homeostatic regulation ^94^, one of its defining features. Only a few genetic screens assessed the effects of sleep loss, even fewer have quantified the dynamics of the processes that accumulate during wakefulness and dissipate during sleep, and none systematically evaluated the negative impact of sleep loss on, for example, cognitive outcome variables. Quantifying the sleep homeostatic process is not trivial. When sleep deprivation is included as part of the phenotyping pipeline, usually a single sleep-deprivation duration is chosen, precluding the empirical quantification of the relationship between time spent awake and the resulting compensatory change in the sleep variable concerned. Instead, sleep variables quantified after sleep deprivation are contrasted to their levels during undisturbed baseline conditions, thereby implicitly assuming that these baseline levels represent what the subject “needs”. Whether baseline levels are indeed the levels that are homeostatically defended seems unlikely, especially under standard laboratory conditions (with little stimulation and challenge), animals might sleep more than required. Moreover, the widely used sleep homeostatic proxy ^95^—EEG delta power—is phenotypically and genetically complex and not all changes observed in this variable can be interpreted as proof of an altered underlying sleep homeostat ^5, 94^.

The concept of sleep homeostasis as a process in which a need for sleep accumulates during wakefulness and decreases during sleep is compatible with a feedback oscillation similar to that underlying circadian rhythms with the important distinction that the resulting oscillation is sleep-wake–driven in the case of the sleep homeostat whereas it is self-sustained in the case of the circadian clock. Although the role of clock genes in circadian rhythm generation is firmly established, their function goes well beyond what their name suggests. One example of this is the notion that they also fulfill a function in sleep homeostasis ^96^. Our recent study demonstrated that the cortical expression of the core clock genes *Npas2* and *Clock* are sleep-wake–driven and not circadian ^97^. Moreover, the same study showed that a short 6-hour sleep deprivation caused a long-term (>48 hours) dampening of the circadian amplitude in the cortical expression of most other clock genes. Such results suggest that in peripheral and especially in extra-SCN brain tissues the clock gene circuitry may be used to track time spent awake and asleep rather than circadian time, which is compatible with the notion that clock genes integrate environmental and systemic cues such as temperature, corticosterone, light, and metabolic state, a number of which change according to sleep-wake state ^96, 98^. Therefore, the clock genes might already provide us a core set of sleep (homeostatic) genes that are preserved mostly from *Drosophila* to humans ^96^.

The approaches we presented can be broadly divided into two complementary approaches: one aimed at discovering naturally occurring allelic variants affecting sleep and the other at discovering genes by inducing mutations (randomly) on a single genetic background. The former seeks to explain the large phenotypic variability present in natural populations. Identifying the underlying genetic variants that have been maintained during natural selection, though challenging, might give insight into molecular pathways upon which evolutionary forces shape variation within and between species as well as insight into the variants determining disease risk. By disrupting single genes, the latter approach seeks to identify causal, large-effect genes to uncover specific molecular or physiological pathways. These genes may or may not vary in natural populations or be very rare. Thus, both approaches are needed to move the field forward.

We already presented an example from the circadian field in which an induced mutation of large effect was phenotypically rescued when expressed on a different genetic background ^31^. Similarly, the effect of major disease risk variants such as for the *Brca1* and *2* genes in hereditary breast cancer is strongly affected by polygenic background ( https://doi.org/10.1101/19013086). Identifying the modifier genes that can offset the monogenic risk effects is obviously of importance in increasing the accuracy of risk assessment and deciding on therapeutic strategies. To systemically explore the effects of genetic background and to discover modifier genes affecting disease risk, established transgenic mouse models of Alzheimer disease have been crossed to a GRP, such as the BXD recombinant inbred panel, to create a resource for experimental precision medicine ^99^. Such combined approaches could also be taken for major-effect genes identified in mutagenesis or knockout screens for sleep to study the epistatic interactions and charting the molecular pathways involved.

Though not the focus of this review, studying intermediate phenotypes (that is, *omics) can aid in identifying genes implicated in sleep and its regulation, especially when combined with genomic information ^5, 76, 77^. For example, sleep-wake–driven changes in transcriptome, metabolome, or phosphoproteome studies led to molecular pathways for sleep homeostasis and key genes therein such as *Homer1*, *Acot11*, and *Sik3* ^5, 17, 72, 100^. Given the enormous number of “genome-wide” data sets that have been accumulated, methods to mine and integrate them become increasingly important. Supervised and unsupervised machine learning methods (for example, ^101– 103^) are being actively developed to better integrate multi-omics datasets and infer causality, and their implementations in the sleep field are ongoing. Finally, the information contained in the DNA alone is insufficient to explain phenotypic variability, and the importance of the contribution of activity or accessibility of non-coding regulatory elements (for example, enhancers, silencers, and promotors) is increasingly acknowledged in the sleep field ^104, 105^ as these were shown to be sensitive to sleep loss with surprisingly fast dynamics ^97, 106^. 

## Abbreviations

BXD, panel of recombinant inbred mouse lines derived from a C57BL/6J (B) x DBA/2J (D) intercross; CC, Collaborative Cross; CFW, outbred population of mice derived from Swiss Webster (W) at Carworth Farms (CF); CRISPR-Cas9, clustered regularly interspaced short palindromic repeats and CRISPR-associated protein 9; DGRP, Drosophila genetic reference population; EEG, electroencephalogram; EMS, the mutagen ethyl methanesulfonate; ENU, N-ethyl-N-nitrosourea; eQTL, expression quantitative trait locus; GRP, genetic reference population; GTEx, Genotype-Tissue Expression; GWA, genome-wide association; IKMC, International Knockout Mouse Consortium; IMPC, International Mouse Phenotyping Consortium; NREM, non–rapid eye movement; QTL, quantitative trait locus; REM, rapid eye movement; RLS, restless legs syndrome; SCN, suprachiasmatic nuclei; SNP, single-nucleotide polymorphism; UAS, upstream activation sequence
